# Abnormalities in A-to-I RNA editing patterns in CNS injuries correlate with dynamic changes in cell type composition

**DOI:** 10.1038/srep43421

**Published:** 2017-03-07

**Authors:** Nurit Gal-Mark, Lea Shallev, Sahar Sweetat, Michal Barak, Jin Billy Li, Erez Y. Levanon, Eli Eisenberg, Oded Behar

**Affiliations:** 1 Mina and Everard Goodman Faculty of Life Sciences, Bar-Ilan University, Ramat Gan 52900, Israel; 2 Department of Developmental Biology and Cancer Research, Institute of Medical Research Israel-Canada (IMRIC), Faculty of Medicine, The Hebrew University, Jerusalem 91120, Israel; 3 Department of Genetics, Stanford University, Stanford, California 94305, USA; 4 Raymond and Beverly Sackler School of Physics and Astronomy and Sagol School of Neuroscience, Tel Aviv University, Tel Aviv 69978, Israel

## Abstract

Adenosine to Inosine (A-to-I) RNA editing is a co- or post-transcriptional mechanism that modifies genomically encoded nucleotides at the RNA level. A-to-I RNA editing is abundant in the brain, and altered editing levels have been reported in various neurological pathologies and following spinal cord injury (SCI). The prevailing concept is that the RNA editing process itself is dysregulated by brain pathologies. Here we analyzed recent RNA-seq data, and found that, except for few mammalian conserved editing sites, editing is significantly higher in neurons than in other cell populations of the brain. We studied A-to-I RNA editing in stab wound injury (SWI) and SCI models and showed that the apparent under-editing observed after injury correlates with an approximately 20% reduction in the relative density of neurons, due to cell death and immune cell infiltration that may account for the observed under-editing. Studies of neuronal and astrocyte cultures and a computational analysis of SCI RNA-seq data further supported the possibility that a reduction in neuronal density is responsible for alterations in the tissue-wide editing patterns upon injury. Thus, our data suggest that the case for a mechanistic linkage between A-to-I RNA editing and brain pathologies should be revisited.

RNA editing is a co- or post-transcriptional mechanism that alters the sequence of an RNA transcript relative to the genomic sequence. The most frequent form of RNA editing is a change in which genomically encoded adenosine (A) is converted to inosine (I) in stem–loop structures within precursor mRNAs. Since inosine is interpreted as a guanosine by the cellular translation machinery, editing has the potential to recode an mRNA and thereby increase the protein repertoire^1^,^2^.

A-to-I editing is catalyzed by ADAR enzymes. Three ADAR gene family members have been identified in vertebrates^3^,^4^,^5^. ADAR1 (ADAR) and ADAR2 (ADARB1) are catalytically active and are expressed in many tissues. The two enzymes have different affinities and overlapping specificities for precursor mRNA targets^6^,^7^,^8^,^9^. ADAR3 (ADARB2), exclusively expressed in the brain, has not shown any catalytic activity so far. *ADAR1* encodes two distinct isoforms: Expression of *ADARp150* (encoding a 150-kDa protein) is induced by interferon and detected mainly in the cytoplasm, and *ADARp110* (encoding a 110-kDa protein) is constitutively expressed and localized exclusively in the nucleus^10^.

Due to the rapid progress in sequencing technologies, a huge number of editing sites have been identified in recent years: several millions in human^11^,^12^,^13^ and thousands in mouse^14^,^15^ and *Drosophila*^16^,^17^,^18^. Most of the editing sites are located in noncoding sequences, and their biological importance remains largely unknown. The vast majority of these sites reside within inverted, usually lineage specific, repeats: the *Alu* element pairs in human^19^,^20^,^21^,^22^ and the B1/B2 elements in mouse^15^. These pairs are likely to form long double-stranded RNA (dsRNA) structures, the optimal targets for ADAR enzymes^6^. These long dsRNA structures may often be deaminated at multiple sites. This type of editing is referred to as hyper-editing^23^,^24^,^25^. The presence of A-to-I editing events in repetitive elements and other hyper-edited transcripts regulates the structure and stability of dsRNA structures and has been recently shown to play an essential role in regulating the innate immune response^26^,^27^,^28^.

Editing sites that reside within the coding sequence and result in modification of the resulting protein (also called recoding sites) are rare. Only a few dozen mammalian conserved ADAR targets have been identified^29^. Such sites typically exhibit high editing levels whereas editing of repeat sequences is generally low but widespread^13^.

A-to-I editing levels change dynamically across developmental stages and under varying conditions: editing was shown to be lower in early stages of development and more abundant as development progress^30^,^31^,^32^,^33^. In particular, A-to-I editing in young neurons was recently shown to be low compared to mature cells^32^. Alterations of A-to-I editing have been reported in different diseases of the central nervous system such as depression, epilepsy, schizophrenia, Alzheimer’s disease, and amyotrophic lateral sclerosis^34^,^35^,^36^,^37^,^38^. Recent studies have demonstrated that there is a reduction in A-to-I editing following spinal cord injury (SCI)^32^,^39^. In some cases, the observed correlations between brain diseases and modified editing have been interpreted as a mechanistic association between the pathology considered and the editing events, their outcome, or their regulation. We reasoned, however, that a change in the cell population could explain the observed change in editing after pathology onset or injury.

Brain tissues are composed of multiple cell types^40^,^41^. Together with neurons, glia and vascular cells are essential for the normal development and function of the central nervous system. Glial cells include astrocytes, microglia, and oligodendrocytes in various states of maturation states. The CNS also includes specialized blood capillaries, composed of specialized endothelial cells and pericytes, that form the blood brain barrier. Under conditions of inflammation, injury, and in many neurodegenerative conditions, immune cells, including monocytes, macrophages, and T and B cells may also infiltrate the brain parenchyma.

In this study, we analyzed RNA-seq data obtained from purified cell populations from the mouse brain. We quantified RNA editing in these cells using various methods and demonstrated that editing levels varied considerably across cell-types. In particular, neurons exhibited the highest editing activity among all major brain cell types. We then used two separated mice cohorts of brain injury models, stab wound injury (SWI) and SCI, to demonstrate a reduction in RNA editing following injury, and argue that the apparent under-editing is accounted for by a reduction in the neuronal density upon injury. Thus, we suggest that the possibility of a change in cell-type composition (e.g., reduced neuronal density) should be taken into account while analyzing any observed change in editing level found in different brain pathologies.

## Results

### Neurons are the major contributors of A-to-I editing activity in the brain

To profile the editing pattern across all major brain cell types, we analyzed previously reported RNA sequencing data obtained from seven mouse brain cell-types (two biological replicate samples of pooled animals for each cell type were sequenced^40^, data accessible through GEO Series accession number GSE52564). Highly purified neurons, astrocytes, oligodendrocyte precursor cells (OPCs), newly formed oligodendrocytes (NFOs), myelinating oligodendrocytes (MOs), microglia, and endothelial cells were obtained by immunopanning with cell type-specific cell-surface antibodies and FACS of transgenically labeled cell populations. Neurons, astrocytes, and endothelial cells were purified at P7, whereas oligodendrocyte-lineage cell isolation occurred at P17 for full collection availability. We excluded one of the microglia samples from our analysis due to low sequencing quality and alignment rates. In addition, we analyzed RNA-seq data generated from three mouse whole cerebral cortex tissue samples that were sequenced in the same study^40^ (data accessible through GEO Series accession number GSE52564), and RNA-seq data from purchased primary neural stem cells (NSCs) isolated from embryonic mouse cortex (E14.5)^42^ (data accessible through GEO Series accession number GSE74643).

**Figure 1:**
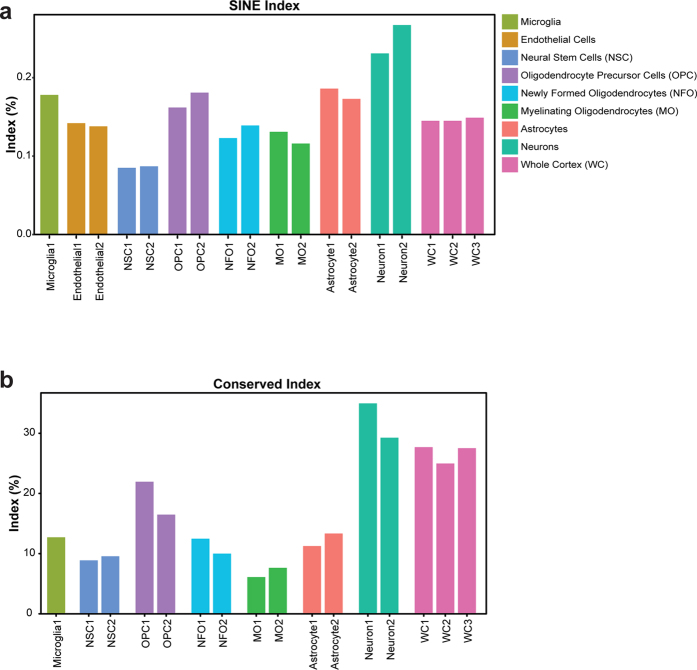
Neurons exhibit elevated editing relative to other brain cell types. Comparison of global editing measures across cell types isolated from cerebral cortex samples (two replicates for each cell type, one microglia excluded) and three whole cerebral cortex samples (where the editing level is the weighted average over all cell types). (**a**) SINE editing index, the weighted editing level over all adenosines in SINEs (**b**) CEI, the weighted editing level over all mammalian conserved sites.

For each cell type or tissue sample, we employed three different A-to-I editing measurement schemes to estimate global editing activity: (i) Since most editing events take place within lineage-specific genomic repeats, we adjusted the human *Alu*-specific editing detection algorithm described by Bazak *et al*.^13^,^43^ to quantify the editing in SINE sequences (see Methods). (ii) A hyper-editing detection tool^23^ was applied to identify heavily edited reads that are often overlooked by standard alignment methods (Supplementary Figure S1a and Methods). Many of the editing events identified by the hyper-editing tool occur within SINEs, and they were merged into the SINE editing index (Fig. 1a). In both global SINE and hyper-editing analysis A-to-G showed higher signal than other possible substitutions (Supplementary Figure S1b). (iii) Finally, we quantified RNA editing activity in each of the conserved mammalian ADAR targets (see list in Supplementary Table S1). These sites, although few in number, are highly conserved, tend to exhibit high editing levels, and are mostly located within genes encoding neurotransmitter receptors or other synapse-related proteins^29^. Thus, their editing is believed to play an important functional role. We calculated a global conserved-editing-index (CEI) for each cell type or tissue sample, representing the weighted average of editing levels over all mammalian conserved sites (Fig. 1b and Methods).

**Figure 2:**
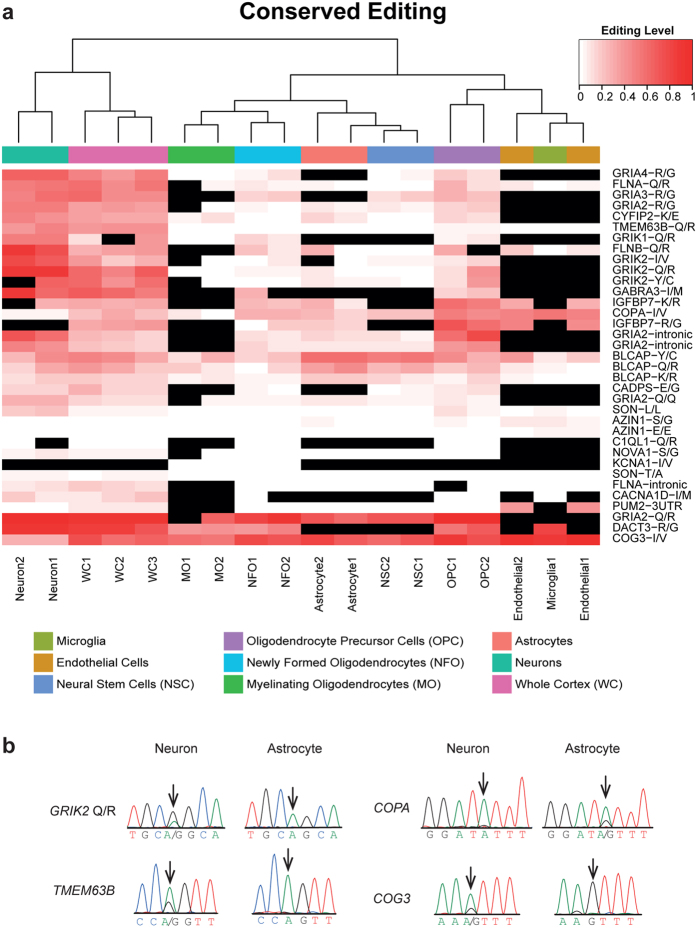
Distinct patterns of RNA editing in mammalian conserved editing sites across brain cells subpopulations. (**a**) The editing profile across the conserved sites, presented for each of the samples analyzed in Fig. 1. If coverage did not exceed 15 reads, editing levels was not calculated (black). Editing sites where this cutoff was not achieved in at least two kinds of cell types were discarded. Note that endothelial and microglia samples are lowly expressed in many of the conserved sites. (**b**) Sanger sequencing validation of differential editing in selected targets using isolated neurons and astrocytes cultures.

Noticeably, all detection schemes applied reveal a considerable variation of editing levels across cell-types, where neurons exhibit the highest editing activity among all major brain cell types (Fig. 1 and Supplementary Figure S1a). A detailed study of editing levels in each of the conserved mammalian sites also demonstrated a clear cell-type dependence (Fig. 2a and Supplementary Table S2), but the pattern of variation was complex. Most sites (including the well-characterized sites in *FLNA, CYFIP2* and the Q/R site in *GRIA2*) were edited more strongly in neurons than in other cell types. However, a few exceptions were observed: For example, levels of editing in *COPA* and *COG3* were higher in glial and vascular brain cell types than in neurons. We validated the differential editing levels of *GRIK2* (at the Q/R site), *TMEM63B, COG3*, and *COPA* using conventional Sanger sequencing of neurons and astrocytes isolated from embryonic and neonatal origin (Fig. 2b). Second to neurons, OPCs demonstrate the highest editing levels, higher than other major glial and vascular brain cell types (further supported by calculation of the conserved index in Fig. 1b). The high editing levels in neurons and subsequently in OPCs cannot be explained by high expression levels of genes harboring mammalian conserved editing sites (Supplementary Figure S2 and Supplementary Table S3).

**Figure 3:**
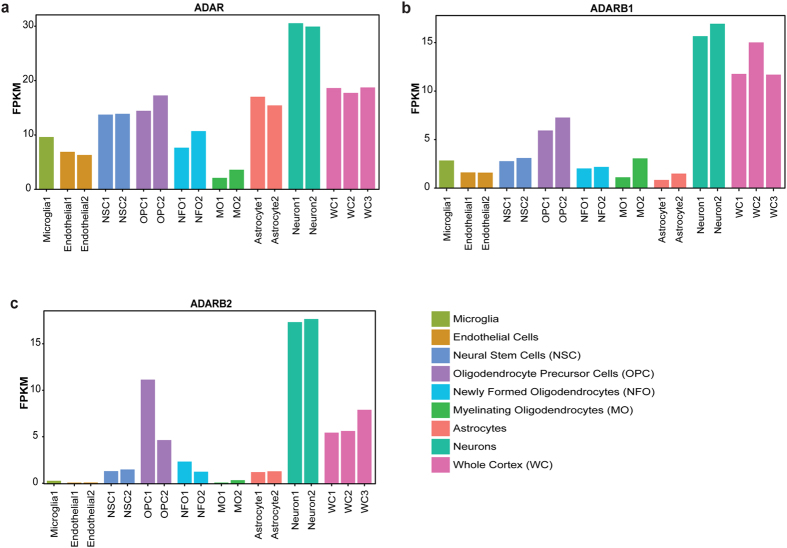
*ADAR* transcripts are over-expressed in neurons. FPKM values for each cerebral cortex cell-type and whole cerebral cortex samples are presented. Neurons over-express (**a**) *ADAR*, (**b**) *ADARB1*, and (**c**) *ADARB2* in comparison to other cell types.

Consistently, the functionally active *ADAR* and *ADARB1* are over-expressed in neurons compared to other brain cell types (Fig. 3a and b). *ADARB2* displays a similar pattern of expression (Fig. 3c). In particular, *ADARB1* expression level across tissues correlates well with editing levels of the conserved sites (Figs 3b and 2a), supporting the notion that conserved sites are commonly edited by ADARB1 and to a lesser extent by ADAR.

As editing levels vary across cell-types, editing levels in whole tissues strongly depend on the relative abundances of cell sub-populations present in the whole tissue. Accordingly, when studying differential editing, one must examine the possibility that changes in the sub-population proportions may be responsible for the observed alterations. We now demonstrate this point by studying the apparent alteration in RNA editing levels in CNS injury models.

### Reduction in RNA editing levels in CNS injury models

A decrease of RNA editing in acute and late responses to SCI was previously reported by Hwang *et al*. and Narzo *et al*.^32^,^39^. Here we focused our attention on the acute phase response to SCI (2–3 days following injury). We also implemented an additional model of CNS injury, cortical SWI, to screen for alterations in RNA editing. We compare our findings to RNA-seq data from SCI obtained by Chen *et al*.^44^ and demonstrate, in both cohorts, a reduction in editing following injury that is affected by dynamic change in tissue cell composition.

**Figure 4:**
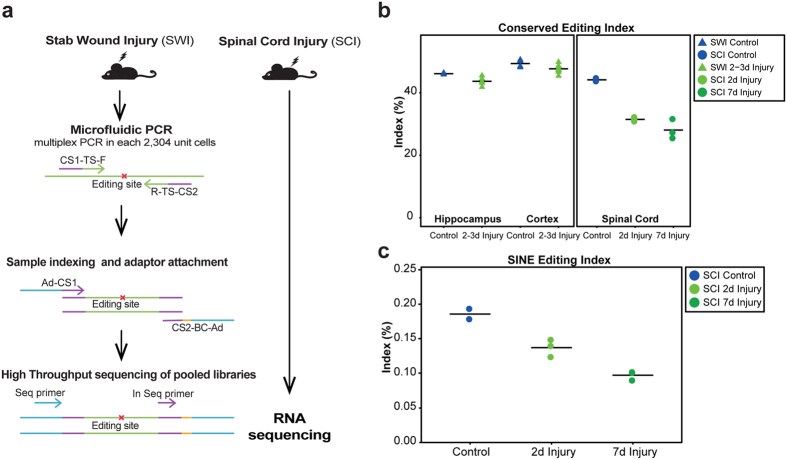
Decrease in A-to-I global RNA editing measures following CNS injuries (SWI and SCI). (**a**) Schematic illustration of study workflow using microfluidics-based multiplex PCR (mm-PCR) coupled with next generation sequencing. TS: target-specific sequence; CS: custom sequence; BC: barcode sequence; Ad: Illumina adaptor sequence; Seq primer: Illumina sequencing primer; In Seq primer: Index sequencing primer. (**b**) Reduction in CEI induced by CNS injuries. A similar reduction in global editing levels at conserved sites was observed for the SWI (triangles) and SCI (dots) models. Each of the SCI data points represents a pool of three mice. (**c**) SINE editing index in control (blue), acute SCI (2d; bright green) and subacute SCI (7d; dark green) samples reveals a decrease in global editing levels following injury.

In the SWI model, injury was directed independently to the cortex tissue on each lateral side of the brain. Two affected cortex and hippocampus samples (the hippocampus was situated directly below the injured cortex region), were collected from each animal 2 to 3 days following injury. Hippocampus was studied as it is known to be hypersensitive to injury of the cortex. Overall, the study included 24 brain samples (8 injury and 4 control for each hippocampus and cortex tissues). We then used the microfluidics-based multiplex PCR (mmPCR) methodology for targeted resequencing^45^ to quantify in high-resolution editing levels in a panel of pre-selected sites, most in non-repetitive regions in the mouse genome (Fig. 4a and Methods).

Of the 189 sites that passed our filters (Supplementary Table S4 and Methods), 165 sites were not conserved. None of these sites have shown significant differential editing between SWI samples and control samples (after multi-testing correction). In contrast, eight of the 24 conserved editing sites examined exhibited differential editing in hippocampus (Supplementary Tables S5 and S6). Six of the conserved sites displayed decreased editing levels upon injury: *FLNA* Q/R, *TMEM63b* Q/R, *CACNA1D* I/M, *GRIK2* Q/R, *GRIK2* I/V, and *BLCAP* Y/C. At two sites, COPA I/V and COG3 I/V, increased editing was induced by SWI. The same trend was observed in the cortex samples, although differences did not reach statistical significance (Supplementary Table S6).

Assessment of the CEI also revealed a reduction in RNA editing activity within the reduced panel of conserved editing sites (Fig. 4b, left panel). In hippocampus, the CEI was significantly decreased following SWI (46.10% ± 0.13% to 43.66% ± 0.37%, Mann-Whitney p-value 0.004), while in cortex, the CEI, similarly to the single sites pattern, was also decreased but did not reach statistical significance (49.34% ± 0.61% to 47.66% ± 0.47%, Mann-Whitney p-value 0.073).

We then analyzed RNA-seq data derived from 24 female mice following contusive SCI^44^ (data accessible through GEO Series accession number GSE45376). Each biological replicate consisted of three mice pooled together: sham control (Control, n = 2) and acute and sub-acute phases of SCI (2 or 7 days after injury, 2d and 7d respectively, n = 3 each). Here too, we observed a reduction in RNA editing activity following injury: The CEI was lower after injury (44.15% ± 0.49% to 31.47% ± 0.39% at 2 days and 28.07% ± 1.82% at 7 days; Fig. 4b, right panel, and Supplementary Table S7), the SINE editing index was reduced (0.186 ± 0.008 in control, 0.138 ± 0.007 at 2d, and 0.098 ± 0.004 at 7d; Fig. 4c), and fewer editing clusters were identified by the hyper-editing detection tool (11.66 ± 1.28 in control, 8.26 ± 0.35 at 2d and 4.65 ± 0.69 at 7d; Supplementary Figure S3). No statistical analysis was performed due to the small sample size. Of note, the results found for acute phase of SCI (2d) conform with those obtained in the SWI model, which is similar in its timeline. The results for the subacute phase (7d), a further stage of injury not tested in SWI, show an even larger reduction in editing activity indexes.

Differences in editing levels in the acute phase of SCI correlated well with the corresponding results in hippocampus and cortex following SWI (Supplementary Figure S4). Six out of the eight conserved sites differentially edited in SWI hippocampus compared to control were found to be in full agreement to the editing alterations observed in the acute phase of SCI (Table 1 and Supplementary Figure S5).

**Figure 5:**
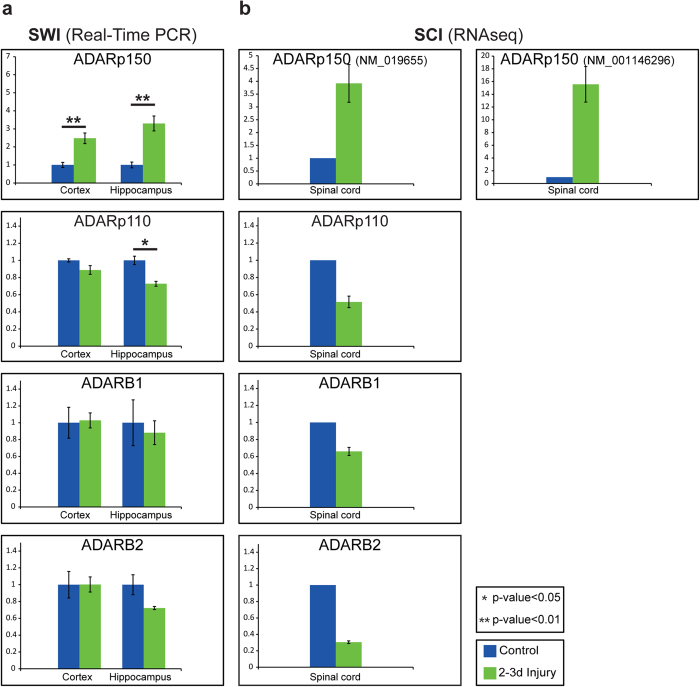
Changes in *ADAR* expression levels following CNS injuries. (**a**) Relative levels of *ADAR* isoforms (*ADARp110* and *ADARp150*), *ADARB1*, and *ADARB2* in control and SWI samples (cortex and hippocampus) were quantified by real-time PCR analysis. The y-axis represents the relative expression compared to the *CHMP2A* reference gene (ΔΔCt method). (**b**) *ADAR*s expression levels in acute SCI samples (2d) based on FPKM values as reported by Chen *et al*. (no statistical analysis, due to the small sample size). Error bars represent one standard error of mean.

Consistently, quantitative real-time PCR showed reduced expression levels of *ADARp110, ADARB1*, and *ADARB2* in hippocampus tissue following SWI (Fig. 5), although only the decrease in *ADARp110* transcript achieved statistical significance (p-value 0.024). In contrast, *ADARp150* transcript level was significantly elevated following SWI both in hippocampus and cortex (p-values 0.004 and 0.0095, respectively, see Fig. 5), which may be indicative of a neuro-inflammatory process consequential to brain injury.

### Reduction in RNA editing following CNS injuries is affected by tissue cell diversity

**Figure 6:**
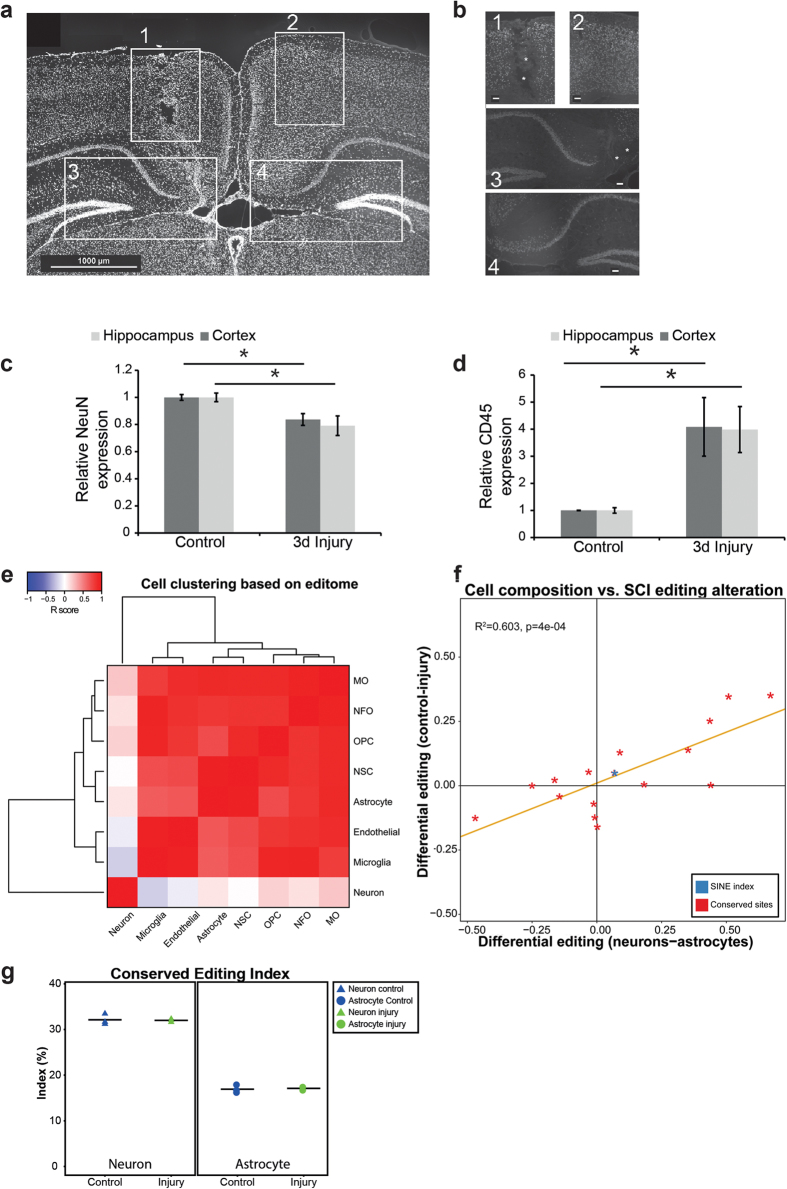
Reduced A-to-I RNA editing in CNS injuries is explained by a dynamic change in cell type composition. (**a**) Representative example of a coronal section of cortex and hippocampus after injury in low magnification, stained with DAPI. Box 1: injured cortex; box 2: cortex on contralateral non-injured side; box 3: injured hippocampus; box 4: hippocampus on contralateral non-injured side. (**b**) The boxed regions shown in panel A stained with anti-NeuN. Stars in boxes 1 and 3 mark sites of neuron loss as a result of the injury. Scale bar, 79 μm. Note that while neurons are lost at the injury site, the total density of cells (based on the DAPI staining) is higher on the injured side as compared to the contralateral side. (**c**) Relative mRNA levels of *NeuN*, a neural marker, measured by real-time PCR analysis (means ± SEM) for cortical and hippocampal tissues 3 days post-injury. (**d**) Relative mRNA levels of *CD45*, an immune cell marker (means ± SEM). **(e)** Brain cells cluster into two distinct groups based on their editome profile: neuronal vs. non-neuronal. Clustering was done based on pairwise Pearson correlation of the editing levels at conserved sites. (**f**) Differential editing upon injury (SCI, 2d vs. control) correlates with the differences in editing between neurons and astrocytes. We included conserved editing sites (red) and the SINE editing indices (blue). (**g**) No change in CEI was observed in cultured neurons and astrocytes following injury in an *in vitro* model.

Changes in RNA editing following injury may follow from reduced editing activity in the cell. Alternatively, they can represent a reduction of the relative abundance of neurons (neuron density) in the tissue at the site of injury. To visualize the cells in proximity to the injury we used DAPI staining. In the SWI model, the densities of cells were similar on injured and non-injured sides of the brain 3 days after injury (Fig. 6a). To visualize the neurons in proximity to the injury we used NeuN staining since all neurons in the cortex and hippocampus express this nuclear marker. The number of neurons was reduced on the injured side compared to the control side of the brain (Fig. 6b), indicating that the neuronal content is lower in proximity to the injury.

To get a more quantitative estimate of neuronal density, we used real- time PCR to estimate the levels of *NeuN*, since based on the NeuN staining intensity (Fig. 6A), injury does not affect the levels of NeuN in individual neurons. Thus, quantification of *NeuN* expression provides a good estimate of the neuronal density in the tested site. We observed a reduction of about 20% in the levels of *NeuN* on the injured relative to the contralateral side (Fig. 6c, Mann-Whitney p-value for cortex 0.0079 and for hippocampus 0.0476). The reduced density of neurons may be due to neuron loss (Fig. 6b) and to an increase in other cell types, such as immune cells that infiltrate the brain paramecia following injury.

To estimate immune cell content we used real-time PCR to quantify *CD45*, a general marker of all immune cells. We observed a ~4-fold increase in *CD45* levels in injured tissue relative to the control (Fig. 6d, Mann-Whitney p-value for cortex 0.0158 and hippocampus 0.0159). Together our results show that the neuronal content of the brain tissue is reduced following injury likely because of neuronal cell death and infiltration of immune cells. Since editing is generally higher in neurons than in other cell types, this change in cell composition results in a reduction in tissue-averaged editing, even though editing in each sub-population is unaffected by injury.

Can the change in neuronal density explain the observed alteration of the editome upon injury ? In order to answer this question we used a simplified computational model that simulates the effect of neuronal density change on the global editing indices. As seen in Fig. 6e, the various cell sub-populations can be broadly classified into neurons and non-neurons based on their editomes. We therefore looked at the differential editing levels of specific conserved sites as well as the global SINE index in neurons and non-neurons, and correlated these differences to the differential editing signal observed when comparing the SCI model (2d) to the control. Strikingly, these two are very well correlated (Pearson R^2^ = 0.603): Editing sites that are highly edited in neurons are under-edited upon SCI, whereas those sites that are edited at low frequency in neurons (such as *COG3* and *COPA*) exhibit elevated levels following SCI (Fig. 6f). This correlation suggests that the complex changes observed in the editome following injury are well explained by a change in the neuronal density rather than to global changes in editing efficiencies.

Furthermore, in order to test directly whether editing levels are modified upon injury within specific cell types, in parallel to the change in tissue content, we established an *in-vitro* model system. We plated highly enriched neuron and astrocyte cultures and grew the cells for a week in culture. The cultures were then mechanically injured, and RNA was collected 24 h following the injury. RNA editing levels were analyzed by the mmPCR methodology as mentioned above. The overall CEI was high in cultured neurons and low in cultured astrocytes (Fig. 6g and Supplementary Table S8), in accordance to the above (Fig. 1b). However, CEI and the editing levels in each culture of differentially conserved edited sites identified in both CNS injuries, was not affected by the injury (Table 2 and Fig. 6g). These results are consistent with the notion that the major contributor to RNA editing changes following injury originates from cell type composition and to a lesser extent, if any, by injury itself.

Taken together, these results indicate that the observed alteration of the editome following injury is a direct result of the change in neuronal density, while editing is modified very little, if at all, within each cell type.

## Discussion

RNA editing in brain tissues has been studied extensively^21^,^30^,^32^,^33^; however, the complexity due to the various types of cells in brain has been largely ignored in the context of editing. Here we show that editing in the brain varies considerably across cell types. Editing frequency is much higher in neurons at most sites evaluated than it is in other cell types. There were exceptions. Some editing sites, such as those in *COPA* and *COG3*, are heavily edited in some cell populations of the brain but, despite high levels of expression, are not edited to a significant extent in neurons.

The cellular making of the brain and spinal cord tissue is complex. During pathological condition it becomes also very dynamic. Under normal conditions the CNS includes both neurons, glial cells and blood capillaries and is largely lacking of most immune cells. Although there is no agreement in the literature regarding the densities of neuronal and non-neuronal cells in the brain, recent data demonstrated a high fraction of neurons in comparison to other cell types (possibly as high as 50%)^41^,^46^. Together with the high editing level in neurons, this means that even a moderate change in neuronal density may have a considerable effect on global RNA editing patterns. As shown in this study, after brain injury there is a significant increase in immune cells and a decrease in the number of neurons near the site of injury. Together, these two processes reduce the density of neurons in the tissue. Since editing occurs at much higher levels in neurons than in other cells of the brain, this results in an apparent reduction of editing for the whole tissue. Moreover, based on this assumption we would predict that sites with low levels of editing in neurons but with high levels of editing in other populations of cells would show an apparent increase in editing. Indeed, in complete opposite to most editing sites we found an increase in editing of *COG3* and *COPA*. As predicted these two genes are edited to a very limited extent in neurons and are highly edited in other populations such as astrocytes and endothelial cells. Thus, the reduced RNA editing detected here and reported by others in other models of injury does not indicate an intrinsic alteration in the regulation or process of RNA editing but is a natural consequence of the changes in the tissue composition. Indeed, our *in vitro* injury model supports the notion that editing is virtually unchanged upon injury in neurons and in astrocytes. Admittedly, use of cultured cells is limited in several ways, and future single-cell RNA-seq studies will be able to give a more exact estimation of editing frequencies in different cell types. Nevertheless, one can already conclude that cell composition has a strong effect on the editing patterns observed while analyzing whole tissues.

Alterations of RNA editing have been reported based on analyses of pathological conditions, many of which are associated with inflammation and loss of neurons. Based on our analysis, it may be suggested that, similar to the case of brain injury, some of these editing alterations are accounted for by changes in neuronal density or other changes in cell-type composition rather than to the deregulation of RNA editing. If this is indeed the case, the evidence for a mechanistic linkage between RNA editing and these pathologies should be revisited. Further, this finding may be relevant to other molecular process, such as transcription and splicing that have been studied under various conditions at whole-tissue resolution.

## Methods

### Mouse brain cell type and SCI RNA-seq data

The RNA sequencing data of purified neurons, astrocytes, microglia, endothelial and various maturation states of oligodendrocytes from mouse cortex (n = 2 for each) together with whole cortex samples (n = 3) was downloaded from the National Center for Biotechnology Information (NCBI) Gene Expression Omnibus (GEO accession number GSE52564)^40^. To this collection, we added RNA sequencing data from purchased primary neural stem cells (NSCs), that were isolated from embryonic mouse cortex (E14.5) (GEO accession number GSE74643)^42^. The SCI data was reported by Chen *et al*.^44^ (GEO accession number GSE45376).

### Quality control and alignment

The quality of the sequence reads was check by FastQC quality control tool^47^ with default parameters. In the brain cell types collection, one microglia sample was excluded from our analysis due to low sequencing quality and alignment rates. Next we ran STAR RNA-seq aligner^48^ to map sequence reads to the mm9 reference genome. Reads that were map to more than one genomic location were filtered out (outFilterMultimapNmax = 1).

### Detection of global editing levels in SINE Elements

This pipeline was applied on brain cell types and SCI RNAseq databases. We adjusted the human *Alu*-specific editing detection algorithm^13^, to screen three mouse SINE subfamilies: B1, B2 and B4. We included in the index calculation reads that failed to align to the genome, but were aligned using the hyper-editing scheme (see below). Similar to the *Alu* editing index, the SINE editing index is defined as the number of ‘G’s in RNA-reads nucleotides that were aligned to genomic adenosines that reside in SINE element, divided by the total number of read-nucleotides that align to SINE adenosine positions. The higher the index, the more editing occurs in SINE elements. Even though most editing sites in SINEs are very weak, thus hard to quantify reliably, the SINE index that averages over many thousands of these sites, provides a robust measure.

### Global editing in hyper-editing clusters

We used the hyper-editing analysis^23^ to obtain another global estimate of RNA editing levels within various brain cell types and injured spinal cord vs. control samples. This pipeline quantifies heavily edited reads (hyper-edited) which fail to align to the corresponding genome using standard alignment tools, and are hence traditionally overlooked. In order to align these hyper-edited reads, we transform all Adenosines to Guanosines in both the unmapped reads and the reference genome and realign them, and then transform back the nucleotide to identify all mismatches. We verify that the resulting mismatches are pre-dominantly A-to-G, and that the identified sites conform to the familiar ADAR sequence signature (G depletion in the nucleotide upstream to the editing site, and excess of G in the downstream nucleotide) (Supplementary Figure S1, panel c and d)^49^. For each sample, the number of hyper-edited reads per million mapped reads is used to quantify the level of hyper-editing. Note that although many of these reads are aligned to SINEs, and their editing activity is already represented by the SINE index, we did not attempt at removing the SINE contribution from the hyper-editing signal, since some reads are only partially aligned to SINEs.

### RNA editing in mammalian conserved editing sites

A list of mammalian conserved editing sites is presented in Supplementary Table S1. Quantification of editing levels in specific sites was performed using REDITools scripts^50^. We considered only mismatches with phred score >25 at the base that was mapped to the editing site position. In the brain cell types analysis, we discarded sites that were not covered by at least 15 reads in at least two kinds of brain cell types. Samples were grouped by their editing levels using average linkage hierarchical clustering. Heatmap clustering was done by the gplots package in R. For the SCI dataset we applied a cutoff of 15 reads in all samples.

The CEI, weighted average of the editing level in all conserved sites, was evaluated for each sample in the brain cell types and the SCI databases. The index is defined as the ratio of the number of A-to-G mismatches (summed over all conserved sites, no cutoffs applied) to the total number of reads in these sites. We excluded the two endothelial samples from this analysis since IGFBP7 was vastly overexpressed in these cells, accounting for >70% of the total FPKM value of all host genes.

### Gene expression analysis

Expression levels of *ADAR* genes and genes containing mammalian conserved editing sites were calculated by the RSEM tool^51^. Values are presented in units of Fragments per Kilo base Of Exon per Million Fragments Mapped (FPKM) to normalize the influences of the sample size and the genes length. For SCI RNAseq database, the FPKM values were downloaded from Chen *et al*.^44^. Hierarchical clustering of expression levels was done using gplots package in R.

### Animal SWI model and tissue preparation

C57B6 mice were purchased from Harlan Laboratories. Animal handling adhered strictly to national and institutional guidelines for animal research and was approved by the Ethics Committee of the Hebrew University of Jerusalem. Cortical injury experiments were performed on mice aged 6–7 weeks. For the injury experiments mice were anesthetized with ketamine/xylazine solution (50 mg/kg ketamine 7.5 mg/kg xylazine in 0.9% NaCl solution). A sterile needle was inserted vertically into the right and left cerebral hemisphere, reaching the skull surface at a depth of 5 mm. The needle was inserted through the cranium 2 mm caudal to the bregma and 1 mm lateral to the midline. The skin incision was closed with sutures. Two and three days following injury mice where sacrificed and tissue biopsy samples 3mm by 3mm of the cortex at the injured epicenter and part of the hippocampus directly beneath the site of injury were frozen.

### DNA and RNA extraction and sample preparation

Total RNA was extracted using Trizol (Invitrogen). From three cortex samples we consecutive isolated genomic DNA and total RNA (NORGEN kit). RNA was treated with DNaseI (Roche) and reverse transcribed into cDNA using iScript™ Advanced cDNA Synthesis Kit (Bio-Rad).

### Immunofluorescence analysis

Brains were fixed in 4% paraformaldehyde, following by incubation in 30% sucrose for at least 24 h. Tissue sections (15 micrometers thick) were incubated for 1 h in a blocking solution consisting of 0.3% Triton X-100, and 5% goat serum. This solution was used for the dilution of both primary (anti-NeuN 1:400, Cell Signaling Technologies, Danvers, MA) and secondary antibodies. Sections were incubated in the primary antibody overnight (16 h). Slides were then washed three times (5 min each) with PBS and visualized with Cy3-labeled secondary antibody and coverslipped. At least seven sections were analyzed for each mouse.

### Neuronal and Astrocyte cell cultures

Day 2 Mouse primary cortical astrocytes were cultured as described previously^52^. Mouse primary cortical neurons were cultured essentially as described previously, with minor modifications^53^. Briefly, cerebral hemispheres were aseptically removed from embryonic day 16 (E16) embryos and the cortices were incubated in 20 u/ml papain for 10 min at 37 °C. Enzyme activity was then stopped by trypsin inhibitor containing 5% BSA. Tissues were then mechanically dissociated and resuspended in Neurobasal medium and B27 supplement. Cells were grown in pre-coated poly-L-lysine 140 cm2 culture plates for seven days. 50% of the medium was replaced on day 3.

### Target amplification and analysis of RNA editing sites using the Fluidigm Access Array microfluidic system

See Supplementary methods.

### Real-Time PCR quantification assays

The relative mRNA quantification of *ADARp110, ADARp150, ADARB1, ADARB2, NeuN* and *CD45* was determined using qRT-PCR on the 7900HT Fast Real-Time PCR System (Applied Biosystems, Foster City, CA). For each tested gene, we used 14 injured samples and 7 or 8 biological control samples (from cortex and hippocampus origin). cDNA sample were tested in triplicates using the PerfeCTa SYBR Green FastMix (Quanta BioSciences, Gaithersburg, MD) using the following specific primers:

ADAR-p110: 5′-GCAGCGTCCGAGGAATCG-3′ and 5′-TAAGACTCCGGCCCCTGTG-3′

ADAR-p150: 5′-CACTATGTCTCAAGGGTTCAGGG-3′ and 5′-CACTTGCTATGCTCATGACTAGGG-3′

ADARB1: 5′-GTCCTGCAGTGACAAGATAGCAC-3′ and 5′-GGTTCCACGAAAATGCTGAG-3′

ADARB2: 5′-GGGGAGCAGCTAATCACCAT-3′ and 5′-TGGAGGTACACAGGCTCGAT-3′

NeuN: 5′-GGTGGAGTTGCTGGTTGTCT-3′ and 5′-GCACAGACTCATCCTGAGCA-3′

CD45: 5′-TAGGCTTAGGCGTTTCTGGA-3′ and 5′-TCGTGCCCAAACAAATTACA-3′

The relative quantification of gene expression levels were normalized to CHMP2A expression level (NM_014453) using the following gene specific primers; 5′-AAGGCCAGATGGATGCTGT-3′ and 5′-CCGCATCAACACAAACTTGC-3′ and the ΔΔCt method was applied^54^. The CHMP2A gene was selected for normalization since its expression was found to be exceptionally uniform across a large panel of tissues based on RNAseq data^55^.

### Sanger sequencing validation

To confirm the editing level obtained by RNAseq and targeted resequencing, PCR was performed using Phusion Hot Strart II High-Fidelity DNA Polymerase (Thermo Scientific) to amplify FLNA, TMEM63B, GRIK2 Q/R, COPA and COG3. The PCR program was set according to manufacture recommendation. Resulting PCR amplicons were purified using PCR Purification kit (Invitrogen) and editing was determined using the reverse primer by Sanger sequencing (Applied Biosystems).

### Brain cell types and CNS injury correlation

We used the SINE editing value together with editing levels of conserved editing sites, followed by pairwise correlation and clustering performed by gplot package in R, to compare the editome of the various cell-types. As the data showed a clear separation between neurons and non-neurons (R > 0.64 without neurons while R > −0.09 when including neurons), we chose the astrocyte as a representative non-neuron cell type, calculated the differences between editing levels in neuron and astrocyte samples, and correlated them (Pearson) to the differential editing signal observed for SCI sample vs. control.

#### Ethical approval

Animal handling adhered strictly to national and institutional guidelines for animal research and was approved by the Ethics Committee of the Hebrew University of Jerusalem.

## Additional Information

**How to cite this article:** Gal-Mark, N. *et al*. Abnormalities in A-to-I RNA editing patterns in CNS injuries correlate with dynamic changes in cell type composition. *Sci. Rep.* **7**, 43421; doi: 10.1038/srep43421 (2017).

**Publisher's note:** Springer Nature remains neutral with regard to jurisdictional claims in published maps and institutional affiliations.

## Supplementary Material

Supplementary Information

Supplementary Tables

## Figures and Tables

**Table 1:** Conserved editing sites differentially edited in CNS injuries.

Editing site	SWI	SCI	p-value SWI	∆SWI	∆SCI (2d)	∆SCI (7d)
*FLNA* Q/R	↓	↓	0.034	−21.47	−34.63	−27.58
*TMEM63B* Q/R	↓	↓	0.034	−9.99	−25.17	−45.22
*GRIK2* I/V	↓	↓	0.034	−6.95	−39.47	−26.37
*GRIK2* Q/R	↓	↓	0.034	−6.95	−25.97	−32.42
*COPA* I/V	↑	↑	0.034	+6.23	+4.23	+14.17
*COG3* I/V	↑	↑	0.041	+8.67	+12.62	+32.93

**Table 2:** Editing levels (%) in primary neurons and astrocytes in an *in vitro* injury model system.

Editing site	Neuron		Astrocyte	
	Injury	Control	Injury	Control
*FLNA* Q/R	7.26 ± 1.01	7.23 ± 0.61	1.96 ± 0.29	1.78 ± 0.28
*TMEM63B* Q/R	31.6 ± 2.25	31.63 ± 0.74	1.26 ± 0.26	1.3 ± 0.08
*GRIK2* I/V	38.74 ± 0.97	38.19 ± 0.8	3.76 ± 1.67	2.74 ± 0.78
*GRIK2* Q/R	69.35 ± 0.74	70.43 ± 1.09	4.68 ± 0.08	2.95 ± 0.5
*COPA* I/V	3.1 ± 0.14	3.29 ± 0.09	18.35 ± 1.81	18.04 ± 0.74
*COG3* I/V	24.69 ± 2.92	22.97 ± 1.25	88.56 ± 3.25	91.95 ± 1.3

Average editing levels and STDEV of differentially conserved editing sites identified in CNS injuries are presented.
