# Supplementary material for: Abnormalities in A-to-I RNA editing patterns in CNS injuries correlate with dynamic changes in cell type composition
Source: Sci Rep. 2017 Mar 7;7:43421. doi: 10.1038/srep43421 (PMC5339895; doi:10.1038/srep43421)
Supplement: Supplementary Information [file srep43421-s1.pdf]

Abnormalities in A-to-I RNA editing patterns in CNS injuries correlate with dynamic changes in cell-type composition

Nurit Gal-Mark[1,+], Lea Shallev[1,+], Sahar Sweetat[2], Michal Barak[1], Jin Billy Li[3], Erez Y. Levanon[1], Eli Eisenberg[4,*] and Oded Behar[2,*]

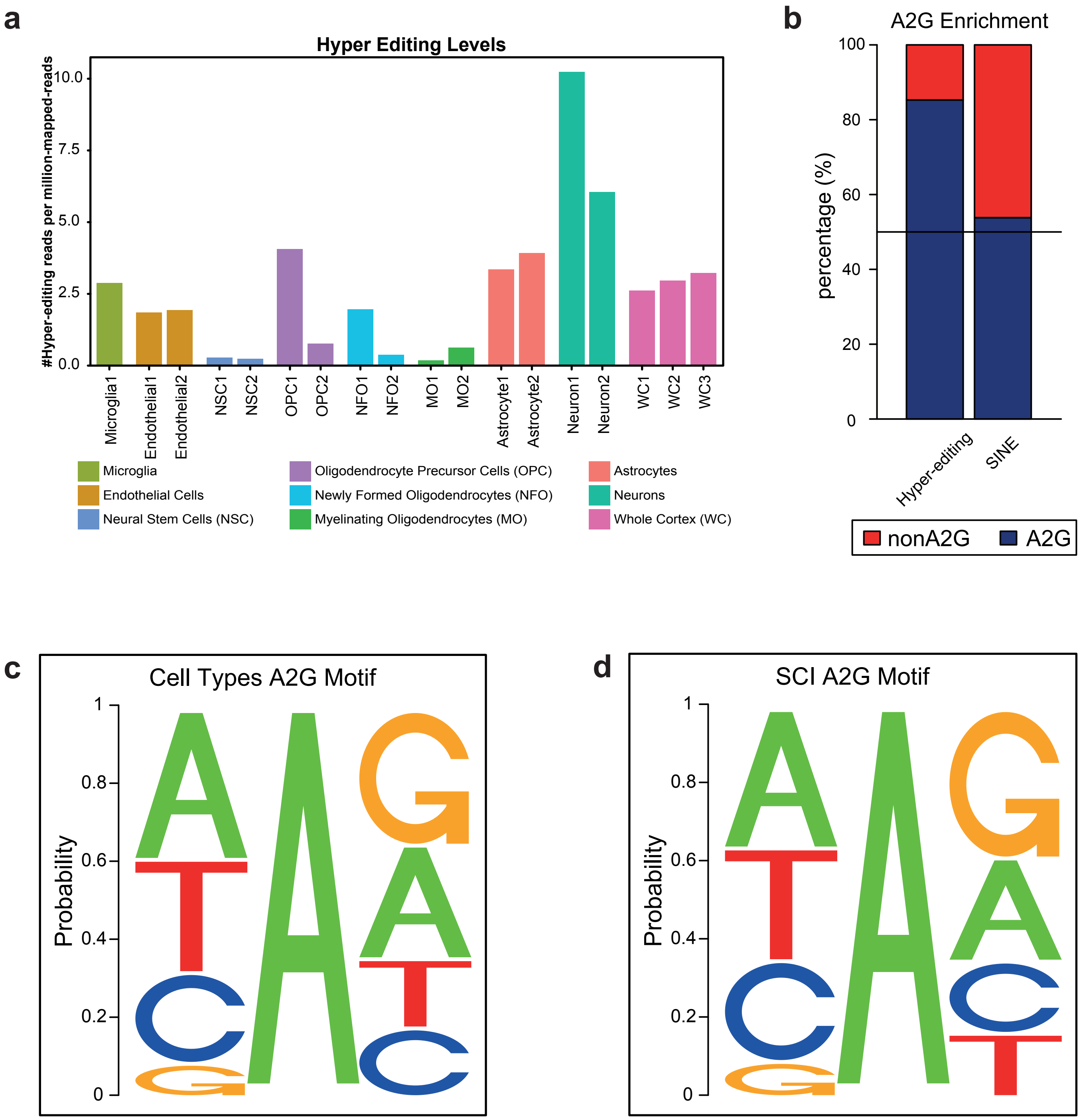

Supplementary Figure S1: Reliable recognition of A-to-I editing sites using SINE and hyper-editing detection schemes. (a) Hyper-editing analysis across the isolated cell types from cerebral cortex samples. The number of hyper-editing reads divided by the number of million mapped reads is presented. **(b)** Percentage of A-to-G mismatches within SINE repeats and hyper-edited clusters versus all other possible substitutions. **(c)** and **(d)** The distribution of nucleotides upstream and downstream of the A-to-G sites detected in the hyper-editing analysis of RNA-seq obtained from (c) brain cell types and (d) SCI. The distributions are characterized by underrepresentation of G at position -1 and overrepresentation of G at the +1 position in accordance with the known ADAR preference.

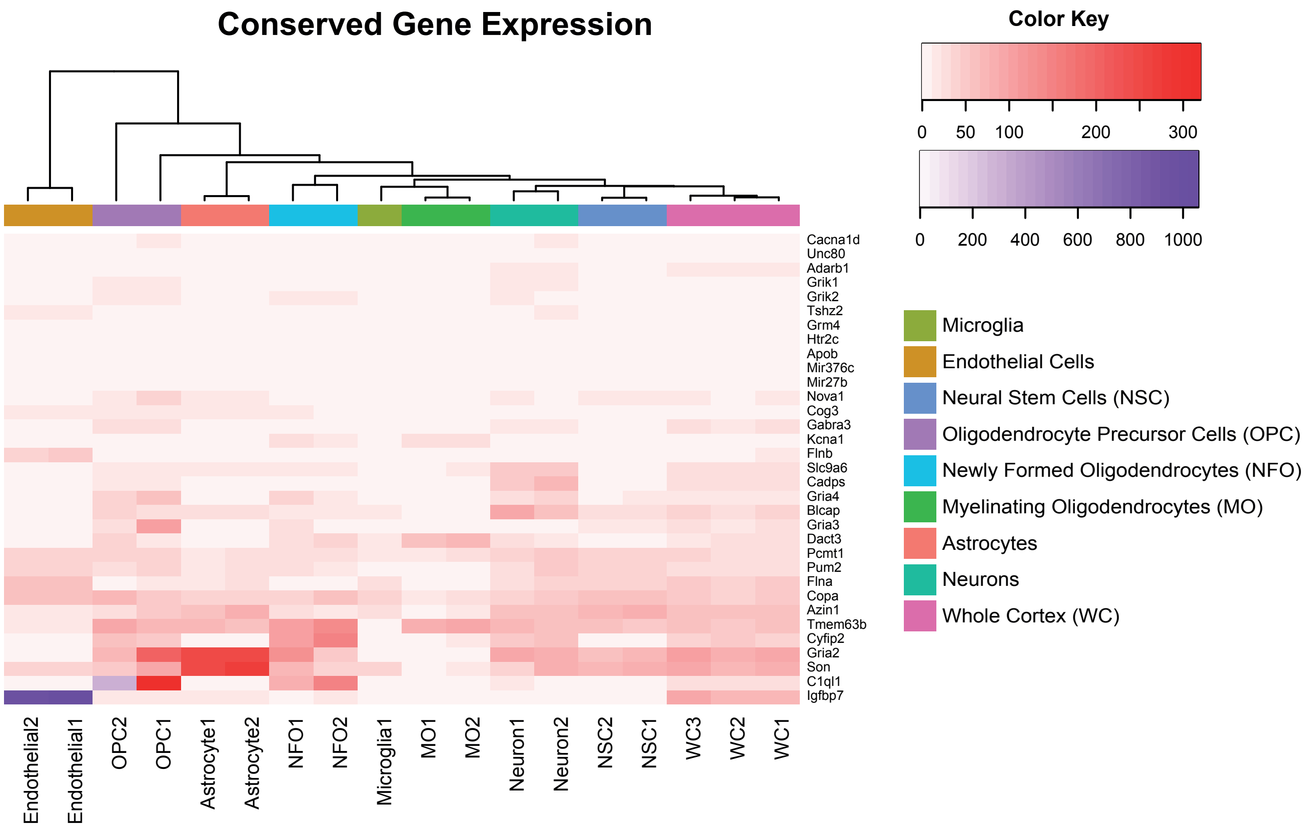

Supplementary Figure S2: Gene expression levels of mammalian conserved editing targets. Expression levels (FPKM) of genes harboring mammalian conserved editing sites in indicated brain cell types.

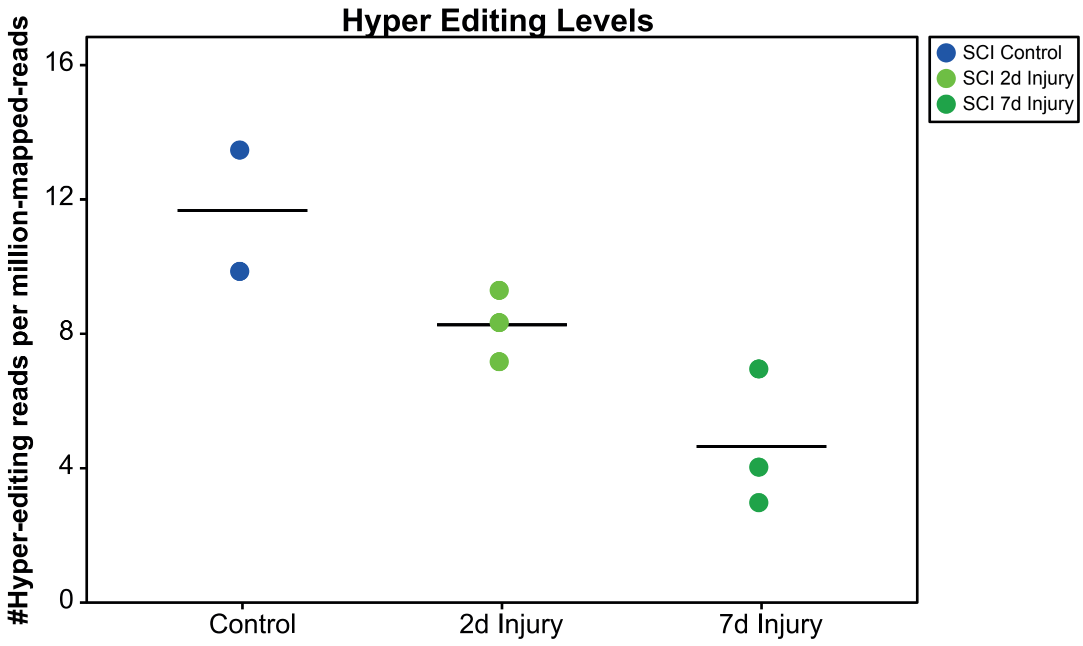

Supplementary Figure S3: Hyper-editing analysis in SCI. A decrease in global editing levels following injury is detected by hyper-editing scheme. Analysis of Control (blue), acute SCI (2d; bright green) and subacute SCI (7d; dark green) samples are presented.

a

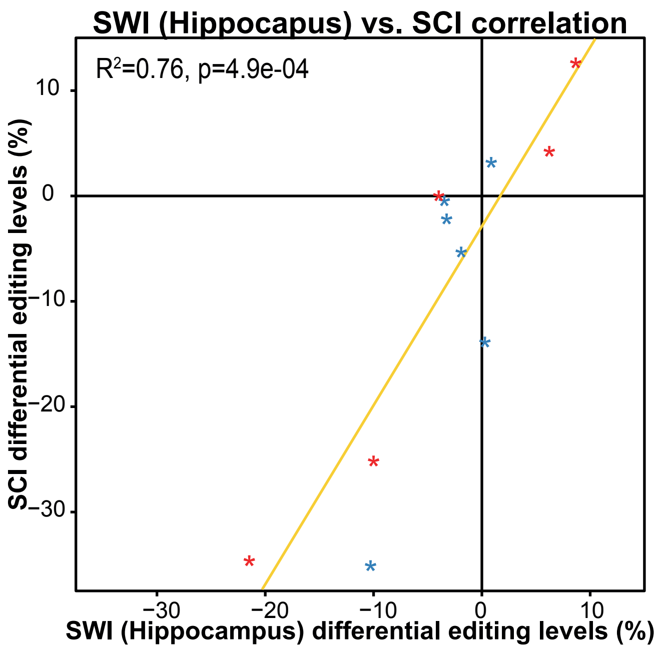

b

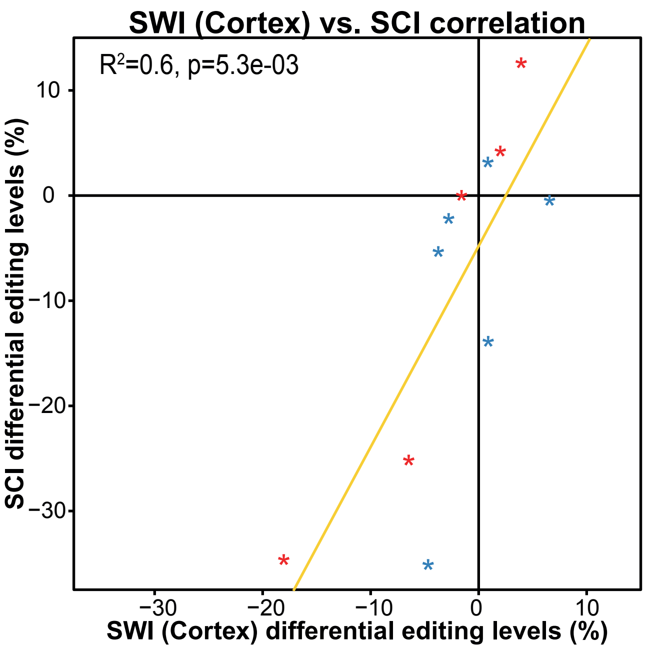

c

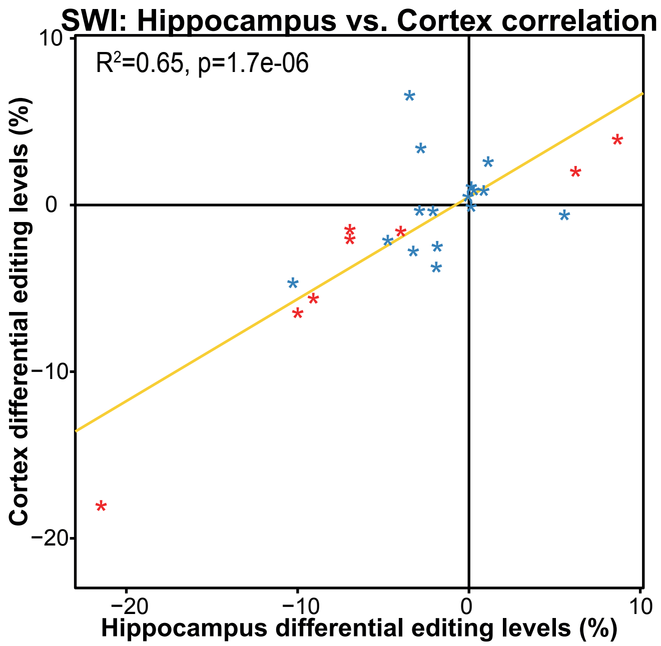

Supplementary Figure S4. Comparison of the two CNS injury models. Differential editing observed in both models is well correlated in comparisons of **(a)** SWI hippocampus vs. acute SCI, **(b)** SWI cortex vs. acute SCI, and **(c)** SWI hippocampus vs. SWI cortex. Each asterisk represents a conserved editing site. Editing sites that exhibited significant differential editing in hippocampus following SWI are indicated in red.

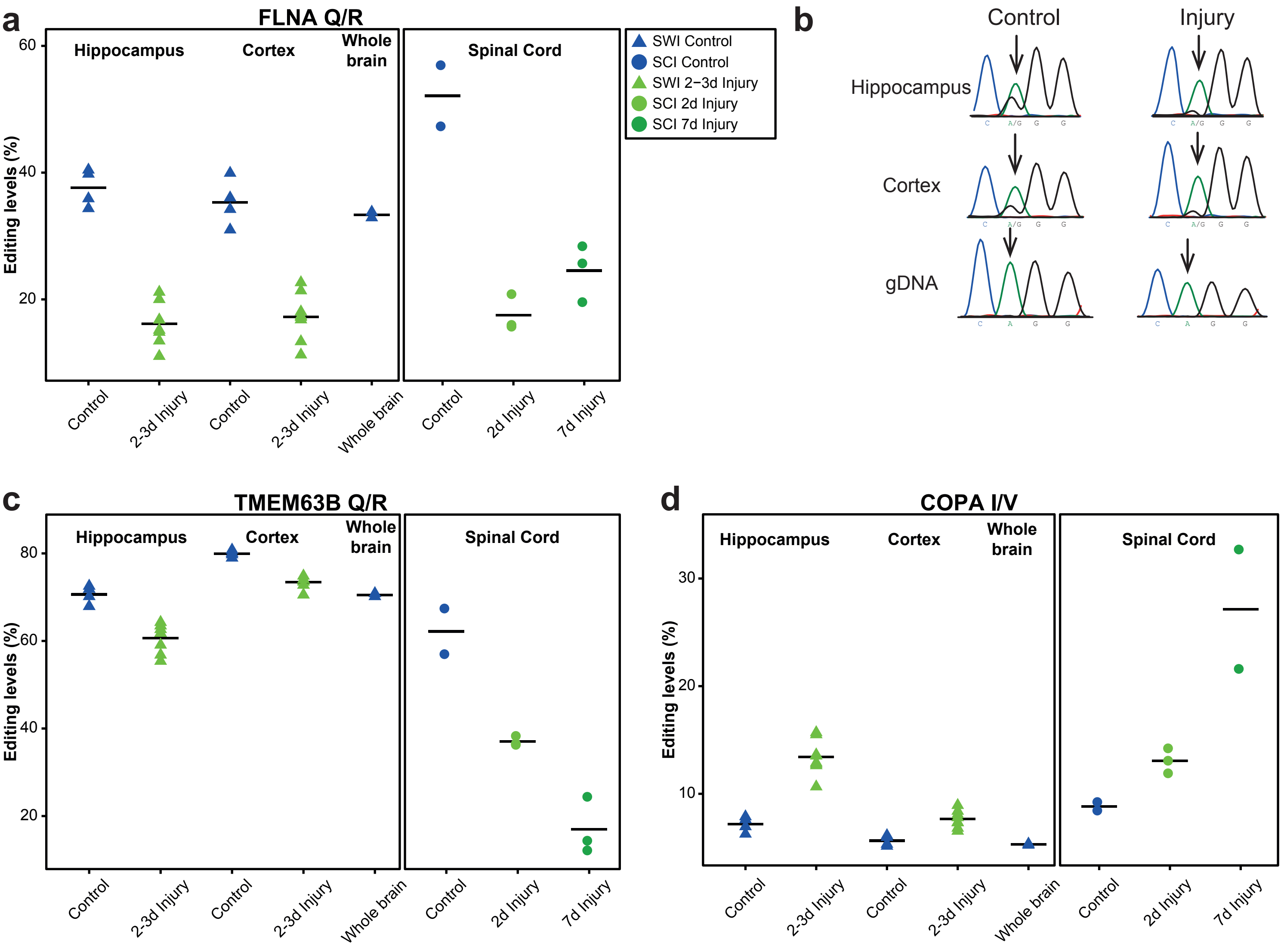

Supplementary Figure S5. Editing alterations at specific sites upon CNS injuries. Differential editing at **(a)** *FLNA* Q/R, **(c)** *TMEM63B* Q/R, and **(d)** *COPA* I/V conserved editing sites in SWI and SCI models based on mmPCR (triangles) and RNAseq (dots). **(b)** Validation of FLNA differential editing using direct Sanger sequencing.

Supplementary Methods

Target amplification of RNA editing sites using the Fluidigm Access Array microfluidic system

To gain high-resolution view of A-to-I RNA editing alterations following SWI, we used microfluidics-based multiplex PCR (mmPCR) methodology for targeted resequencing built on the Fluidigm Acess Array system which enables the parallel analysis of multiple samples across focused editing locus. Deep sequencing provides high per-base coverage to allow reliable quantification of sites tested. This methodology was previously described by Zhang et al [45] and was scaled up to quantify editing levels in 535 A-to-I RNA editing sites (Supplementary Table S9) chosen from the RADAR database (Rigorously Annotated Database of A-to-I RNA editing) [54]. cDNA samples underwent preamplifycation before the microfluidic multiplex PCR, using FastStart High Fidelity PCR system (Roche): 120ng of cDNA sample was added to 5 ul of primer pool (173 nM per primer) and FastStart High Fidelity pre-sample mix containing: 1.5 µl of 10X FastStart High Fidelity Reaction Buffer with MgCl2, 0.75 µl DMSO [5%], 0.3µl 10mM PCR Grade Nucleotide Mix [200 µM], 0.15 µl of FastStart High Fidelity Enzyme Blend and PCR grade water to complete to 15µl. We used the following PCR program for preamplification: 95°C 10 min, 2 cycles of 95°C 25 sec, 60°C 4 min and 72°C 30 sec and 13 cycles of 95°C 25 sec and 72°C 4 min. Following preamplification, amplified products was purified using PureLink PCR Purification Kit (Invitrogen).

The Fluidigm access-array chip (IFC) was loaded with mouse RNA editing primer pairs (~10-12 multiplex primer pairs in each primer inlet) along with cDNA samples

(in sample inlets). Thermal cycling was performed according to manufacturer's recommendations.

Sequencing adaptor and barcode addition

Resulting PCR amplicons were labeled by a subsequent PCR with barcode sequences specific for each sample: 1.0 μl of the PCR products harvested from the IFC was diluted by 1:110 and added to 2 ul of universal forward primer (2 uM) , 2 ul of a specific reverse primer (2 uM)) and 15 μl of pre-sample mix prepared with the Roche FastStart High Fidelity PCR System (2 μl of 10X FastStart High Fidelity Reaction Buffer wo/Mg, 1 μl DMSO [5%], 0.4 μl 10mM PCR Grade Nucleotide Mix [200 μM], 3.6 μl 25mM MgCl2 [4.5mM], 0.2 μl of FastStart High Fidelity Enzyme Blend and 7.8 μl of PCR grade water). The following PCR program was used: 95°C 10 min, 11 cycles of 95°C for 30 sec, 60°C for 30 sec, and 72°C for 1 min, and 72°C 5 min. All pools were then combined at equal volume, purified using PureLink PCR Purification Kit (Invitrogen) and extracted from 4% agarose Egel (Invitrogen). Finally, next-generation sequencing was employed to achieve deep coverage allowing for accurate measurement of RNA editing levels per genomic locus. Over 20 million 150 pair-end reads were received.

Data analysis of SWI targeted sequencing library

The reads were aligned to the RefSeqs of mm9 using BWA version 0.7.4 and the mem option and removing reads that aligned to more than one location in the reference with the same score (accounting for duplication of gene variants in the same location). To cover locations that were not covered in the RefSeq data we also aligned the read to the mouse genome (mm9) with BWA, removing reads that aligned to more than one location in the genome with the same score. Next, we used an in-house

script to count the different nucleotides at each of the editing positions tested. For each location we used reads that had phred score over 20 for the nucleotide in the location. Conserved editing index was calculated as mentioned previously for the brain cell types and SCI databases.

To allow for accurate quantification of editing levels we included in our analysis only editing sites for which at least 80% of injured and control samples had a coverage depth of at least 500 reads. We next discarded sites that exhibited very low (0-2%) editing levels in all samples. 189 RNA editing sites met the above criteria (S4 Table), 24 of them were mammalian conserved sites (S5 Table). For each editing site, in both groups, differences in editing level between injured and normal samples (cortex and hippocampus) were evaluated using the Mann Whitney test followed by 5% FDR multiple-testing correction (Benjamin Hochberg adjustment for multiple comparisons, applied separately to conserved and non-conserved editing sites).
